# Think laterally: horizontal gene transfer from symbiotic microbes may extend the phenotype of marine sessile hosts

**DOI:** 10.3389/fmicb.2014.00638

**Published:** 2014-11-19

**Authors:** Sandie M. Degnan

**Affiliations:** Marine Genomics Lab, School of Biological Sciences, The University of QueenslandBrisbane, QLD, Australia

**Keywords:** lateral gene transfer, marine invertebrate, symbiotic bacteria, extended phenotype, host–symbiont interactions

## Abstract

Since the origin of the animal kingdom, marine animals have lived in association with viruses, prokaryotes and unicellular eukaryotes, often as symbionts. This long and continuous interaction has provided ample opportunity not only for the evolution of intimate interactions such as sharing of metabolic pathways, but also for horizontal gene transfer (HGT) of non-metazoan genes into metazoan genomes. The number of demonstrated cases of inter-kingdom HGT is currently small, such that it is not yet widely appreciated as a significant player in animal evolution. Sessile marine invertebrates that vertically inherit bacterial symbionts, that have no dedicated germ line, or that bud or excise pluripotent somatic cells during their life history may be particularly receptive to HGT from their symbionts. Closer scrutiny of the growing number of genomes being accrued for these animals may thus reveal HGT as a regular source of novel variation that can function to extend the host phenotype metabolically, morphologically, or even behaviorally. Taxonomic identification of symbionts will help to address the intriguing question of whether past HGT events may constrain contemporary symbioses.

## ANIMAL–MICROBIAL SYMBIOSES PROVIDE AMPLE OPPORTUNITY FOR HGT

It is increasingly apparent that all sessile marine animals live in intimate association with a diversity of viruses, prokaryotes and unicellular eukaryotes, many of which may function as beneficial symbionts at some or all stages of the animal life cycle (Douglas, 2010; McFall-Ngai et al., 2013). This is not surprising given that animal–microbial interactions have been shaped by more than 600 million years of evolutionary history, traceable back at least to a common ancestor of all modern animals that lived in an ocean of microbes. This long and constant association has provided ample opportunity for all manner of interactions to evolve between kingdoms. The growing number of documented cases of astonishingly intimate symbioses between animal hosts and bacterial symbionts in particular (reviewed in McFall-Ngai et al., 2013), facilitated frequently by vertical transmission, almost certainly represents just the tip of the iceberg. The field of aquatic microbiology is well-poised to benefit from this growing appreciation of animal–bacterial interactions (e.g., Dubilier et al., 2008; Hentschel et al., 2012) by drawing upon the huge diversity of sessile marine animals and their equally diverse symbionts, coupled with lateral thinking about the nature of the interactions that might occur between them. In doing so, we have the opportunity to apply knowledge of animal–microbe interactions to fundamental concepts in biology, and to marine biotechnological problems and applications. There is hope even that investigations in this area “may inform our understanding of diseases like cancer” (Robinson et al., 2013).

Querying the nature and extent of animal–microbe interactions can increasingly be done at the level of the genome. Genomes clearly reflect the deep, shared ancestry between animals and microbes; all three domains of life share approximately one third of their genes, notably including many that encode central metabolic pathways (Domazet-Loso and Tautz, 2008). Beyond this, however, we are becoming increasingly aware of diverse ways in which the genomes and genome products of animal hosts and their microbial symbionts can interact. Host–symbiont interactions revealed to date include critical roles for bacteria in marine invertebrate host nutrition, defense, reproduction, and development (McFall-Ngai et al., 2013). The sharing of gene products impacts directly on the ecological versatility of the host, because the co-opting of a more diverse genetic repertoire present in its bacterial partners (Lapierre and Gogarten, 2009) allows a host to rapidly expand its metabolic potential. Intracellular bacterial symbionts, for example, often encode proteins for metabolic capabilities that are lacking in the animal host, such as synthesis of essential amino acids, photosynthesis, or chemosynthesis (Unson and Faulkner, 1993; Dubilier et al., 2008; Venn et al., 2008; Wilson et al., 2014). Some marine invertebrates that feed on algae are even able to maintain symbioses with photosynthetically active algal plastids and use their photosynthate directly as a food source (Rumpho et al., 2011).

A less broadly appreciated outcome of host–symbiont interactions is the potential through deep evolutionary time for novel functions to be conferred via inter-kingdom horizontal (lateral) gene transfer (HGT), broadly defined as “non-genealogical transmission of genetic material from one organism to another” (Goldenfeld and Woese, 2007). Although most animal genes that can be assigned as homologs of microbial genes are unambiguously derived by descent (Domazet-Loso and Tautz, 2008), there are an increasing number of cases that instead seem to have originated via HGT (Hughes and Sperandio, 2008; Keeling and Palmer, 2008; Boto, 2014; Schönknecht et al., 2014). The full extent to which HGT has occurred across the animal kingdom is currently unknown and the concept of HGT is not yet a mainstream paradigm in the fields of animal evolution and ecology. Recent technical advances, however, now mean that genomic (and proteomic, metabolomic) resources are rapidly becoming available for an increasing number and diversity of animal hosts and their microbial symbionts. For the first time, there is the promise of comprehensively exploring the way in which microbial genomes, past and present, can shape those of their animal hosts via both shared gene products and HGT. Better understanding of these kinds of host–symbiont crosstalk will also facilitate the proper orchestration of these symbiotic relationships in culture.

Horizontal gene transfer could be another way for symbionts to significantly affect host morphology, physiology, or even behavior, in manner akin to the ‘extended phenotype’ proposed by Dawkins (1982) to describe the effects of parasites on their animal hosts. The aim of this perspective is to provide a framework for investigating the role of HGT of protein-coding genes in the evolution and ecology of marine sessile invertebrates. Many marine invertebrates transmit their symbionts via the egg to the early embryo (Douglas, 2010; Schmitt et al., 2011), which could increase the potential for HGT into germ cells that are yet to be differentiated. This potential is increased even further in animals that present no germline barrier to HGT because they do not establish a dedicated germline early in development (Zhaxybayeva and Doolittle, 2011); this applies especially to sponges (Phylum Porifera), whose cells remain pluripotent throughout adult life (Ereskovsky, 2010). In addition, the life cycle of many marine invertebrates includes budding or excision of pluripotent somatic cells, which provides another opportunity for genetic exchange and subsequent vertical transmission of acquired sequences (Boto, 2014).

In recent years, the number of confirmed cases of HGT into animal genomes has been steadily growing as interest is rekindled and growing databases of genomic resources make evidence more robust (reviewed by Hotopp, 2011; Schönknecht et al., 2014). In a recent review on the subject (Boto, 2014), more than half of the reported cases involved marine sessile invertebrates, albeit still in small numbers from just three phyla (Porifera, Cnidaria, and Chordata).

## HOW CAN HGT EVENTS BE IDENTIFIED WITH CONFIDENCE?

The difficulty of unambiguously identifying HGT events is well-known. Early reports of acquisition of bacterial genes by the human genome, for example, were received with excitement but later shown to be erroneous (Salzberg et al., 2001). Importantly, these early falters served to demonstrate that “surrogate” methods to detect HGT (Ragan et al., 2006), based primarily on sequence similarity, are hopelessly insufficient unless followed up at least by phylogenetic analysis. Phylogenetic incongruence has sometimes been accepted as a gold standard for demonstrating HGT, but has two major limitations. First, deep ancestry can mask evidence of a bacterial heritage if genes continue to evolve in their new genomic context, meaning that only recent HGT events likely retain sufficient phylogenetic signal to be detected (Keeling and Palmer, 2008). Second, and even more critical in this age of genomics, phylogenetics alone cannot discriminate between foreign genes present in an animal genome due to contamination (from symbionts or other sources) and those present due to HGT. Both scenarios result in a phylogenetic distribution of the gene of interest that does not reflect the known evolutionary relationships of the species in which it is found.

Compelling identification of an HGT-acquired gene thus requires at a minimum: (i) phylogenetic evidence that the candidate gene is more closely related to foreign than to animal genes; (ii) genome data showing the candidate gene assembles into a contiguous stretch of DNA with neighboring genes unambiguously of animal origin; and (iii) gene sequence revealing metazoan-like compositional traits, including presence of introns, GC content and codon usage. The second of these requires, of course, the availability of a sequenced and assembled animal genome; the more complete the assembly, the more confident the HGT identification. Where possible, gene expression data showing active transcription of candidate genes in animal cell nuclei can enormously strengthen a case, and also addresses the issue of whether or not the HGT-acquired gene is active in its new genomic context, as discussed below.

## WHAT ARE THE IMPORTANT QUESTIONS ONCE HGT-ACQUIRED GENES HAVE BEEN IDENTIFIED?

Only HGT-acquired genes that are functionally integrated into the animal host genome have the potential to affect host phenotype. Functional integration does not always happen, and should not be assumed based solely on presence of the non-animal gene in the animal genome. Some of the best-studied animal germline symbionts – the *Wolbachia* parasites of arthropods and nematodes – have produced several cases of strong evidence for HGT, but interestingly only some of which have resulted in functional genes in the recipient animal genome (Blaxter, 2007; Ioannidis et al., 2013). Evolutionary theory predicts that successful integration of a transferred gene requires both that it is active in the recipient genome and that it is maintained in that genome over evolutionary time (Blaxter, 2007). To fully explore HGT thus requires answering multiple questions.

### WHAT EVIDENCE EXISTS THE HGT-ACQUIRED GENE IS FUNCTIONAL?

Interplay between genome-encoded metabolic pathways of hosts and symbionts is likely to reflect contemporary ecological demands and must necessarily involve only functional gene products (e.g., Dubilier et al., 2008; Wilson et al., 2014). The same is not true for HGTs, which may reflect deep evolutionary consequences of past ecologies and thus may not presently be functional, even if they have been at some time in the past (Hotopp, 2011). Alternatively, HGT-acquired material that is currently non-functional could potentially be co-opted into novel functions in the future. Further complicating this issue, a transferred sequence may only be partial, or may start accruing mutations immediately upon incorporation into a host genome, rendering it non-functional or tending towards pseudogenization from the start.

Demonstrating active transcription of an HGT-acquired gene, using transcriptomics or gene-specific reverse transcriptase-PCR, both now quite routine methodologies, can provide the first line of evidence relatively simply and cheaply. Transcriptomic approaches in particular are accessible even in species lacking extensive genomic resources (Hofmann and Place, 2007). Evidence that a gene is transcribed and available as an mRNA for translation also demonstrates that it has the necessary *cis*-regulatory module/s to be integrated into an existing animal gene regulatory network. Perhaps less routine, but ultimately more compelling, is the demonstration a gene product beyond an mRNA, which is best achieved by proteomics approaches (Hartmann et al., 2014). Substantial evidence can be drawn bioinformatically by showing that the coding sequence contains all elements necessary for a functional protein (e.g., Gladyshev et al., 2008). Careful examination of coding sequence should also reveal evidence of pseudogenization, which is a demonstrated fate of some HGT-acquired genes (e.g., Kondrashov et al., 2006; Gladyshev et al., 2008; Campbell et al., 2012; Strese et al., 2014). Where possible, spatial expression data of gene transcripts or gene products can determine in which tissues or cells an HGT-acquired gene is active, which can go part way toward predicting possible function of that gene.

One caveat of transcriptomic and proteomic approaches is that mRNA or protein detected as present could feasibly be derived from symbiotic or contaminating bacteria that were present in the original biological material. Specific methodologies that target only polyadenylated transcripts largely protect against the detection of bacterial-derived mRNAs in transcriptomic approaches, but proteomics approaches require a clear isolation of animal from bacterial cells. Where there is doubt about the presence of confounding bacterial cells, spatial expression data showing transcripts confined to animal cells provides strong evidence that the transcript originates from the animal, rather than the bacterial, genome (e.g., Gladyshev et al., 2008). A second caveat is a gene may not be active at all stages of an animal life cycle, or in all tissues, so that biological sampling should encompass multiple life history stages and multiple tissue types to optimize one’s chances of detecting active transcription or translation.

### DO HGT-ACQUIRED GENES TEND TO HAVE COMMON FUNCTIONS?

Might there be particular characteristics of genes that make them more likely to be integrated into the recipient genome post-transfer? Here I exclude consideration of transposable elements, which are often horizontally transferred (see Chapman et al., 2010 for a marine invertebrate example) but which are outside of the scope of this perspective. Genes that encode for a function that is of benefit to the host may be subject to positive selection, thus increasing their chance of stable integration. In particular, interactions between intracellular bacterial symbionts and their animal hosts include sharing of metabolic capabilities (Dubilier et al., 2008; Venn et al., 2008; Rumpho et al., 2011), so we might reasonably ask whether HGT-acquired genes might also tend to be of a metabolic nature. It is unclear whether gene products derived from extracellular symbionts as metabolic add-ins (and used by the animal for defense, for example) can actually be incorporated into animal cells, or even whether this is required for genuine metabolic interplay. HGT, on the other hand, provide a means for gene products to be generated directly inside the animal cells, and thus always accessible to those cells. An interesting extension of this consideration is to ask whether genes involved in host–symbiont metabolic interplay in ecological time might be predisposed to acquisition via HGT through evolutionary time.

In bacterial–bacterial systems, HGTs that functionally integrate into the recipient genome do indeed very often involve genes encoding metabolic enzymes (Ochman et al., 2000). However, the more critical factor appears to be that transferred genes are more likely to be integrated if they encode proteins with low network connectivity, regardless of their function (Jain et al., 1999; Cohen et al., 2011). The small number of currently reported cases of HGT between bacteria and aquatic sessile invertebrates do not yet strongly support one scenario over the other, but the fact that operational genes such as metabolic genes tend to have less connectivity than informational genes (Jain et al., 1999; Cohen et al., 2011) points to their increased likelihood of horizontal transfer.

Instances of metabolic gene transfers include bacterial genes acquired by the genome of (i) the sea anemone *Nematostella vectensis*, specifically genes involved in glyoxylate (Kondrashov et al., 2006) and shikimic (Starcevic et al., 2008) pathways; (ii) the ascidian *Ciona intestinalis*, specifically a cellulose synthase gene (Nakashima et al., 2004); and (iii) the freshwater *Hydra magnipapillata*, specifically genes encoding sugar-modifying enzymes (Chapman et al., 2010). A similar number of cases exist, however, of transfers involving non-metabolic genes. These include transfer into (i) the octocoral mitochondrial genome of a gene encoding a MutS protein with putative mismatch repair activity (Bilewitch and Degnan, 2011); (ii) the sponge genome of a gene encoding a biomineralization-related protein apparently involved in biocalcification (Jackson et al., 2011); and (iii) the genome of cnidarians *Nematostella* and *Hydra* of bacterial pore-forming toxin genes of the aerolysin family (Moran et al., 2012).

These latter two cases are of particular interest in the context of commonality of function of HGTs, because both cases reflect similar events in multiple eukaryote taxa. With respect to the sponge biomineralization-related gene, Ettensohn (2014) has recently proposed multiple, independent HGT origins for Msp130 proteins, also implicated in biomineralization, into the genomes of several invertebrate deuterostome and one protostome (molluscs) clade. In each recipient animal lineage, the transferred msp130 genes appear to have undergone independent, parallel duplications (Ettensohn, 2014), suggestive of positive selection. Similarly, aerolysin toxin genes appear to have been independently acquired via HGT not only by cnidarians, but also by other animals such as insects, and even other eukaryotes such as plants and fungi, where they likely function in predation and defense (Moran et al., 2012). Whether these multiple, independent acquisitions reflect strong positive selection on the acquired gene, possibly coupled with their occurrence as genes with low network connectivity, remains to be determined.

A very different, and especially fascinating, instance of non-metabolic HGTs involves eukaryote-like proteins (specifically ankyrin-repeat proteins) putatively transferred *from* a sponge host *to* the bacterial symbionts; these appear to facilitate manipulation by the symbiont of host cell behavior to the benefit of the bacterial symbiont (Nguyen et al., 2014). This, of course, is completely consistent with the role of HGT between bacterial symbionts and their hosts resulting in an extended phenotype, and future outcomes of this research will be exciting to watch. This example also is relevant to the next question posed below.

### DO HGT-ACQUIRED GENES TEND TO HAVE A TAXONOMIC ORIGIN THAT REFLECTS CONTEMPORARY SYMBIONTS?

Our ability to address this question depends upon (i) the comprehensiveness of bacterial genome databases that permit assignment of HGTs to a specific taxonomic source, and (ii) knowledge of the taxonomy of contemporary symbionts of the recipient animal host. Data in both of these areas is growing rapidly, so that any patterns that do exist should emerge within the next few years. At present, for many of the demonstrated cases of HGT described above, the taxonomic source of the transfer is currently unknown. One reason for this, as exemplified by the biomineralization gene transferred into sponges (Jackson et al., 2011), might be that the event happened so long ago that phylogenetic signal of the source has been eroded. In other cases (e.g., *Hydra*; Chapman et al., 2010), the transfers appear attributable to diverse bacterial phyla, with no enrichment for bacterial relatives of present-day symbionts. In contrast, the shikimate pathway genes identified in *Nematostella* as HGTs from bacteria can be traced phylogenetically to a *Tenacibaculum*-like (Flavobacteria) origin; *Nematostella* appears to host a bacterial symbiont of the same taxonomy, which is present even in early life history stages (Starcevic et al., 2008).

This question is important for two reasons. First, it speaks to the temporal and spatial stability of host–symbiont relationships, in that HGT events reflecting current relationships suggest those particular taxonomic associations have persisted over evolutionary time. Where possible, adding a temporal component to the evolutionary source of the transfer can indicate how long ago the gene may have been transferred (what last common ancestor) and thus how long it has persisted. This returns to the question of longevity as demonstration of genuine integration into the recipient genome, and is best served by HGTs for which evidence in multiple animal lineages permits tracing back to a last common ancestor. Second, it addresses whether animals may be choosy in their source of HGT, even if only as a consequence of accessibility to bacterial DNA. If the phylogenetic source of HGT-acquired genes reflects taxonomy of contemporary symbionts, might this suggest constraint on associations? Could HGT events resulting from symbioses deep in the evolutionary past constrain the taxonomic associations possible in the ecological present? Symbioses between arthropod and nematode hosts and their alpha-proteobacterial *Wolbachia*, which have resulted in multiple HGT events (Blaxter, 2007; Le et al., 2014), will no doubt lead the way in how to investigate these interesting considerations.

## CONCLUDING REMARKS

Given the temporally very long, and spatially very close, associations between marine invertebrate animals and bacteria, it is entirely feasible that HGT from bacterial to marine animal genomes is much more prevalent than currently appreciated. That marine animal–bacterial symbioses are both ubiquitous and simultaneously exclusive strongly suggests that host and symbionts carefully choose their partners, rather than relying on random associations (Douglas, 2010; McFall-Ngai et al., 2013). Might both of the same things be true for HGT? The exact nature of the sophisticated conversation that must be required for animal and bacterial partners to assess, respond to and regulate each other currently is unknown, but a number or players are emerging. Among these are innate immune gene pathways (e.g., Franzenburg et al., 2012; Degnan, 2014), eukaryote-like domains (e.g., Nguyen et al., 2014), quorum-sensing (e.g., Zan et al., 2012), and nitric oxide (e.g., Perez and Weis, 2006). Could HGT be another possibility? Could HGT provide a means for bacteria to gain some control over animal function in a manner that ultimately serves both the symbionts and thus also the host? Might HGT in this context be a means for constraining future symbionts to bacteria of particular taxa?

In insects and vertebrates in particular, evidence is mounting for anatomical, cellular, and molecular determinants that act during early development to prepare progeny for their lifetime of interactions with microbes (reviewed in Pradeu, 2011; Ezenwa et al., 2012). Simultaneously, there is a growing understanding of the diverse mechanisms by which microbes act directly as agents of post-embryonic development (reviewed in McFall-Ngai et al., 2013). The integration of genes horizontally transferred from bacterial symbionts into animal host genomes is a potentially potent mechanism by which bacteria can further influence their host environment to ensure their own persistence through evolutionary time. Increasing availability of genomes for marine invertebrate hosts and their bacterial symbionts now allows us to comprehensively address this question for the first time.

## Conflict of Interest Statement

The author declares that the research was conducted in the absence of any commercial or financial relationships that could be construed as a potential conflict of interest.
